# Bladder Embryonal Rhabdomyosarcoma Among Children: A Descriptive Overview From Saudi Arabia

**DOI:** 10.7759/cureus.23904

**Published:** 2022-04-07

**Authors:** Mohammad A Alghafees, Ziyad Musalli, Meshari A Alqahtani, Ghadah I Alhussin, Ahmed Alasker

**Affiliations:** 1 College of Medicine, King Saud Bin Abdulaziz University for Health Sciences, Riyadh, SAU; 2 Urology, King Abdulaziz Medical City, Riyadh, SAU

**Keywords:** rare cancers, pediatrics, saudi children, rhabdomyosarcoma, bladder cancer

## Abstract

Introduction

Although rhabdomyosarcoma (RMS) is the most common type of soft-tissue sarcoma seen in the pediatric population, it is rarely located in the bladder. This study aims to provide a descriptive overview of bladder rhabdomyosarcoma among children in Saudi Arabia.

Methods

This retrospective cohort study included all children diagnosed with embryonal rhabdomyosarcoma from January 1, 2008, to December 31, 2017. Frequency and percentage were used to display the categorical variables and a mean and standard deviation for the continuous variables. Data were collected from the Saudi Cancer Registry (SCR).

Results

A total of 16 patients were detected. Most of the patients (43.8%) were toddlers (1-3 years) and males (87.5%). Most of the tumors were multifocal (100%), well-differentiated (43.75%), and localized (43.75%). The mortality rate was 12.5% with a diagnosis to death interval of 1.26 + 0.46 years. The incidence pattern of bladder rhabdomyosarcoma fluctuated across the years. The highest incidence of bladder rhabdomyosarcoma (0.17) per 1 million was observed in 2012 while the lowest incidence (0.03) per 1 million was observed in 2015.

Conclusion

We concluded that tumor presentation in early childhood is associated with a better prognosis. Moreover, males are predominantly affected by this tumor. Through our study, we tried to fill the knowledge gap regarding the descriptive statistics of bladder RMS in Saudi children. We believe that it would add significant value to the existing literature and help in better understanding the disease.

## Introduction

Rhabdomyosarcoma (RMS) is the most common type of soft-tissue sarcoma seen in the pediatric population. Soft tissue sarcomas account for approximately 7% of all malignancies in children and adolescents, 40% of which are classified as rhabdomyosarcoma [1]. However, they are rare, representing only 3% to 4% of pediatric cancers overall. RMS can develop in any part of the body; however, it usually begins in the soft and connective tissue of the genitourinary tract. The exact pathogenesis of rhabdomyosarcoma is unclear, but it's believed that the tumor originates from cells called rhabdomyoblasts, which are normally responsible for developing skeletal muscle [2]. Studies show that this type of malignancy is more common in children than adults with age peaks from two to four years old, or from ages 15 to 19, with a higher incidence in males compared to females [2-3]. Lower urinary tract symptoms, such as hematuria, dysuria, frequent urination, and urgency to urinate, are usually seen in patients with rhabdomyosarcoma of the bladder. However, some cases may be asymptomatic and identified incidentally [4].

Histologically, RMS is classified into two major subgroups, embryonal RMS (ERMS), which accounts for 60%-70% of the cases, and alveolar RMS (ARMS) accounting for 20%-30% only [3]. The etiology of RMS is still unknown. However, many studies have linked multiple genetic and environmental factors such as paternal cigarette smoking, advanced maternal age, radiation exposure in the uterus, maternal antibiotic use, and maternal recreational drug use. Neurofibromatosis and Li-Fraumeni syndrome, which are associated with inherited gene defects, has been also linked with RMS [1].

There has been a noticeable lack of studies done in Saudi Arabia to correlate important variables such as risk factors, prevalence, incidence rate, and age group of Rhabdomyosarcoma of the bladder in pediatric patients. Thus, our understanding and knowledge of this disease are limited. This study aims to provide a descriptive overview of bladder rhabdomyosarcoma among children in Saudi Arabia.

## Materials and methods

This retrospective cohort study included all patients diagnosed with embryonal rhabdomyosarcoma from January 1, 2008, to December 31, 2017. Patients who were diagnosed with metastatic bladder tumors were excluded from the study. The data were collected from the Saudi Cancer Registry (SCR), which collects tumor data from all private, military, and Health Ministry hospitals in Saudi Arabia through five regional offices. The variables were grouped according to the year of diagnosis, gender, age, marital status, region and nationality, tumor site of origin, tumor histological subtype, tumor behavior, tumor grade, tumor extent, tumor laterality, the basis of the diagnosis, and survival. Data analysis was performed with the Statistical Package for the Social Sciences (SPSS) version 23.0 (IBM Corporation, Armonk, NY). Frequency and percentage were used to display the categorical variables and mean and standard deviation for the continuous variables.

## Results

A total of 16 patients were detected. Table 1 displays the socio-demographic profile of the patients. As for the age, seven (43.8%) were toddlers (1-3 years), five (31.3%) were preschool children (3-6 years), two (12.5%) were school-aged children (6-12 years), and two (12.5%) were adolescents (12-18 years). The minimum age was one, the maximum was 16, and the mean age of patients was 4.75 + 4.86. As for gender, 14 (87.5%) were males and two (12.5%) were females. As for nationality, 13 (81.3%) were Saudi and three (18.8%) were non-Saudi. As for the place of residence, five (31.3%) were living in the central region, one (6.3%) was living in the eastern region, one (6.3%) was living in the northern region, four (25%) were living in the western region, and five (31.3%) were living in the southern region.

**Table 1:** Socio-demographic profile of the participants (n = 16)

Demographical Characteristics	n	%
Age		
Toddler (1 - 3 years)	7	43.8
Preschool (3 - 6 years)	5	31.3
School age (6 - 12 years)	2	12.5
Adolescent (12 - 18 years)	2	12.5
Gender		
Male	14	87.5
Female	2	12.5
Nationality		
Saudi	13	81.3
Non-Saudi	3	18.8
Place of Residency		
Central Region	5	31.3
Eastern Region	1	6.3
Northern Region	1	6.3
Western Region	4	25
Southern Region	5	31.3
Age		
Minimum	1.00	
Maximum	16	
Mean	4.75	
Standard Deviation	4.86	

Table 2 present the tumor profile. As for the location of the tumor, all 16 (100%) patients had multifocal tumors. As for the grade of tumors, seven (43.75%) had grade I tumors (well-differentiated), five (31.3%) had grade II tumors (moderately differentiated), two (12.5%) had grade III tumors (poorly differentiated), and two (12.5%) had grade IV tumors (undifferentiated anaplastic). As for the extension of the tumor, seven (43.75%) had a localized tumor, five (31.3%) had a tumor with regional direct extension, two (12.5%) had a tumor with regional lymph node and direct extension, two (12.5%) had a tumor with distant metastasis, and one (6.3%) did not have documentation about the extension. As for the lateralization, all 16 patients (100%) had an unknown lateralization status. As for the base of diagnosis, all 16 patients (100%) were diagnosed through histology of the primary tumor. As for the year of diagnosis, one (6.3%) was diagnosed in 2008, 0 (0%) was diagnosed in 2009, three (18.8%) were diagnosed in 2010, 0 (0%) was diagnosed in 2011, five (31.3%) were diagnosed in 2012, 0 (0%) was diagnosed in 2013, two (12.5%) were diagnosed in 2014, one (6.3%) was diagnosed in 2015, two (12.5%) were diagnosed in 2016, and two (12.5%) were diagnosed in 2017.

**Table 2:** Tumor profile (n = 16)

	n	%
Location		
Multifocal	16	100
Grade		
Grade I (well-differentiated)	7	43.75
Grade II (moderately differentiated)	5	31.3
Grade III (poorly differentiated)	2	12.5
Grade IV (undifferentiated anaplastic)	2	12.5
Extension		
Localized	7	43.75
Regional: direct extension	5	31.3
Regional: lymph node and direct extension	2	12.5
Distant metastasis	2	12.5
Base of diagnosis		
Histology of primary tumor	16	100
Year of diagnosis		
2008	1	6.30
2010	3	18.80
2012	5	31.30
2014	2	12.50
2015	1	6.30
2016	2	12.50
2017	2	12.50

**Figure 1:**
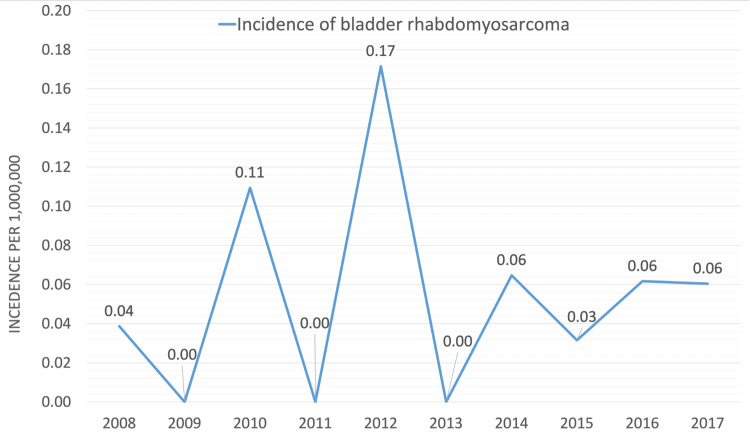
Incidence of bladder rhabdomyosarcoma per 1,000,000 throughout the years

Figure 1 illustrates the incidence of bladder rhabdomyosarcoma per 1,000,000 across the years. The pattern of bladder rhabdomyosarcoma was fluctuating across the years. The highest incidence of bladder rhabdomyosarcoma (0.17) per 1 million was observed in 2012 while the lowest incidence (0.03) per 1 million was observed in 2015. No incidence of bladder rhabdomyosarcoma was registered in 2009, 2011, and 2013.

**Figure 2:**
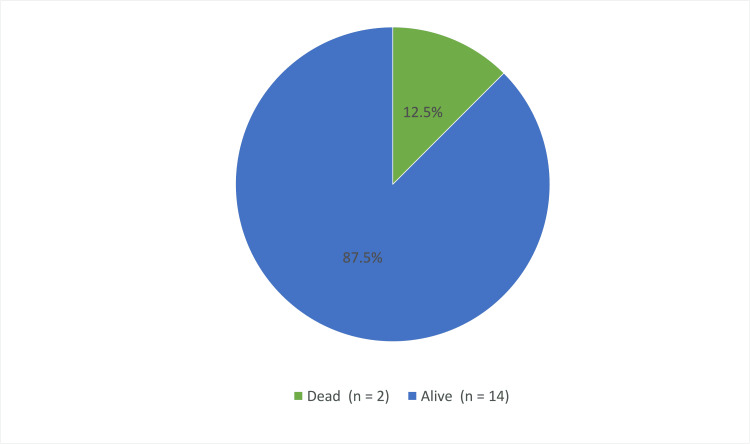
Mortality rate among patients

Figure 2 demonstrates the patient's status. Fourteen (87.5%) were alive while two (12.5%) had passed away. The two patients that died had passed away secondary to cancer.

Table 3 shows the interval in years from diagnosis to death among the patients who died from cancer. The first patient died after 0.94 years of diagnosis (343 days) while the second patient died after 1.59 years of diagnosis (579 days). The mean interval from diagnosis to death was 1.26 + 0.46 years.

**Table 3:** Diagnosis to death interval (n = 2)

Interval From Diagnosis to Death in Years	
The first patient	0.94
The second patient	1.59
Mean	1.26
Standard Deviation	0.46

## Discussion

Rhabdomyosarcomas (RMS) are malignant soft tissue tumors. They originate from immature cells and myogenic satellite cells that form striated skeletal muscle. Nevertheless, RMS can arise in other locations (eg. urinary bladder) where the skeletal muscle is not typically found. It presents as small spindled and round cells with variable eosinophilic cytoplasms on a microscopic level and cellular with features of a small round cell tumor on a cytological level [5].

The head and neck region, genitourinary tract (GU), and extremities are thought to be common sites of primary disease [6]. Stein et al. reported that approximately 13%-20% of all RMS cases are located in the GU region, most commonly in the bladder and prostate [7].

Our study showed a descriptive overview of embryonal rhabdomyosarcomas among children in Saudia Arabia. Embryonal rhabdomyosarcoma is more common in young children than in adults. Among the extracranial solid tumors of childhood, RMS is the third most common neoplasm after neuroblastoma and Wilms' tumor [8]. Approximately 350 new cases are diagnosed in the United States each year, and the annual incidence in children, adolescents, and young adults under the age of 20 is 4.3 cases per one million [9]. RMS follows bimodal age distribution with peaking in early childhood and adolescent age groups. The mean age of the patients included in our study was 4.75 + 4.86 years. In a study done in Turkey, comprising eight patients with bladder/prostate rhabdomyosarcoma, authors reported a mean age of 2.79 years [10]. Similarly, Schreiber and his colleagues mentioned about two cases of embryonal rhabdomyosarcoma in Japan with the age of one and three years [11].

As far as gender predilection is concerned, our study revealed that RMS predominantly affects males. We had 14/16 (87.5%) male and 2/16 (12.5%) female patients in our study. Although not as different as in our study, Agarwala et al. also reported that these tumors are slightly more common in males than in females (1.3-1.4 : 1) [8].

RMS usually arises in the trigone of the bladder and invades the surrounding tissue, presenting as a painful or painless mass. The tumor recurs frequently and metastasizes to the regional lymph nodes, lungs, or liver. As shown in our study, a majority (9/16) of the patients had a direct extension, regional lymph node spread, or distant metastasis. On the other hand, 7/16 patients had localized tumors. Like in our case, the main symptoms of the disease reported by Lauro et al. were hematuria, dysuria, and, more generally, bladder dysfunction [12].

The mortality rate in RMS is highly related to age, location, and histology. Alterio and his colleagues reported the highest five-year survival rate in children aged one to four years, which was 77% [12-13]. It can also be seen in our study that the patients who died belonged to later age groups.

Our study has some limitations because of the small number of the study group. Besides, our study included a heterogeneous group of patients in the cohort. We believe that a more comprehensive retrospective study with a larger data size would be more helpful in better understanding the demographic characteristics, mortality rates, management, and prognosis of embryonal rhabdomyosarcoma among Saudi people. Finally, issues of underreporting, SCR not supplying the data in a way that allows presenting it in the TNM classification, and not including important parameters, such as treatments used, remains an issue that has been highlighted in previous publications utilizing the registry [14].

## Conclusions

Embryonal rhabdomyosarcomas are malignant tumors of mesenchymal origin. They can involve the urinary bladder and present as a painful or painless mass with hematuria, urinary urgency, or frequency. Among the pediatric population in Saudi Arabia, we concluded that tumor presentation in early childhood is associated with a better prognosis. Moreover, males are predominantly affected by this tumor. Through our study, we tried to fill the knowledge gap regarding the descriptive statistics of bladder RMS in Saudi children. We believe that it would be of significant value to the existing literature and help in better understanding the disease.

Registries are urged to include prominent parameters, such as presenting symptoms, the treatment used, and prominent risk factors like the consanguinity of parents, which could be linked to the occurrence of such tumors among children, especially in KSA. Also, further studies on the discovered mutations in genes such as CTNNB1, BCOR, FBXW7, and others may provide targets for novel treatments in patients with rhabdomyosarcoma.
